# TFAP2C facilitates somatic cell reprogramming by inhibiting *c-Myc*-dependent apoptosis and promoting mesenchymal-to-epithelial transition

**DOI:** 10.1038/s41419-020-2684-9

**Published:** 2020-06-25

**Authors:** Yuan Wang, Shuang Chen, Qingyuan Jiang, Jie Deng, Fuyi Cheng, Yi Lin, Lin Cheng, Yixin Ye, Xiaolei Chen, Yunqi Yao, Xiaomei Zhang, Gang Shi, Lei Dai, Xiaolan Su, Yong Peng, Hongxin Deng

**Affiliations:** 1 State Key Laboratory of Biotherapy and Cancer Center, West China Hospital, Sichuan University and Collaborative Innovation Center for Biotherapy, 610041 Chengdu, Sichuan P.R. China; 2 Department of Obstetrics, Sichuan Provincial Hospital for Women and Children, Chengdu, P.R. China; 3 Laboratory Animal Center, Sichuan University, Chengdu, P.R. China

**Keywords:** Cell adhesion, Cell death, Pluripotent stem cells

## Abstract

Transcription factors are known to mediate the conversion of somatic cells to induced pluripotent stem cells (iPSCs). Transcription factor TFAP2C plays important roles in the regulation of embryonic development and carcinogenesis; however, the roles of *Tfap2c* in regulating somatic cell reprogramming are not well understood. Here we demonstrate *Tfap2c* is induced during the generation of iPSCs from mouse fibroblasts and acts as a facilitator for iPSCs formation. Mechanistically, the *c-Myc*-dependent apoptosis, which is a roadblock to reprogramming, can be significantly mitigated by *Tfap2c* overexpression. Meanwhile, *Tfap2c* can greatly promote mesenchymal-to-epithelial transition (MET) at initiation stage of OSKM-induced reprogramming. Further analysis of gene expression and targets of *Tfap2c* during reprogramming by RNA-sequencing (RNA-seq) and ChIP-qPCR indicates that TFAP2C can promote epithelial gene expression by binding to their promoters directly. Finally, knockdown of *E-cadherin* (*Cdh1*), an important downstream target of TFAP2C and a critical regulator of MET antagonizes *Tfap2c*-mediated reprogramming. Taken together, we conclude that *Tfap2c* serves as a strong activator for somatic cell reprogramming through promoting the MET and inhibiting *c-Myc*-dependent apoptosis.

## Introduction

Reprogramming somatic cells to a pluripotent state can be achieved through transient ectopic expression of pluripotency transcription factors (Yamanaka factors), *Oct4*, *Sox2*, *Klf4*, and *c-Myc* (OSKM)^1–3^. The resulting iPSCs hold significant promise as tools for individualized treatment and regenerative therapy^4–6^. However, the derivation of iPSCs is likely a stochastic event, resulting in very low efficiency (~0.1% in humans and ~1.0% in mice) while being time-consuming^1,7^. Indeed, global transcript and protein profiling analysis of intermediates during reprogramming have shown that donor cells undergo a series of phased transitions before reaching the pluripotent state. This process is initiated by the reduction of somatic genes, MET, inhibition of apoptosis and cellular senescence pathways, followed by the upregulation of pluripotency genes, X-chromosome reactivation, telomere elongation and acquirement of the epigenetic characteristics of pluripotent cells^8–10^. In this setup, exogenous factor expression is required for at least 1–2 weeks to establish the endogenous transcriptional network that sustains pluripotency independent of transgene expression. Studies have extensively investigated the molecular mechanisms underlying the formation of iPSCs and sought to identify novel factors that are able to overcome the bottleneck and improve this inherently inefficient process.

*Tfap2c* (also known as *AP2γ*, *Tcfap2c*) belongs to the AP2 transcription factor family, which plays important roles in the regulation of proliferation, cell-cycle control, apoptosis, embryonic development as well as carcinogenesis^11,12^. Mouse *Tfap2c* is expressed in both extraembryonic and embryonic tissues and displays multiple functions in trophectoderm formation, neural crest induction and terminal epidermal differentiation^13,14^. Moreover, *Tfap2c* is required for the survival of the mouse embryo, *Tfap2c* deficient leads to mouse embryonic lethality at approximately embryonic day (E)7.5, which may be attributed to defective placental development^15^. Previous research also revealed the critical roles of *Tfap2c* in trophoblast stem cells (TSCs) maintenance and human primordial germ cells (hPGCs) development^16–18^. However, the roles of *Tfap2c* in regulating somatic cell reprogramming and human naïve pluripotency were not reported until recently^19–21^. Transcriptional analysis of poised iPSC intermediates uncovers *Tfap2c* is important for the acquisition of pluripotency^19^. More importantly, fibroblasts could be reprogrammed into iPSCs by a novel combination consisted of *Gata3*, *Eomes*, *Tfap2c*, *c-Myc*, and *Esrrb*^20^. Despite this, any additional mechanisms accounting for the roles of *Tfap2c* in regulating somatic cell reprogramming are not well understood.

Here we show that *Tfap2c* can greatly promote the generation of iPSCs. Mechanistically, *Tfap2c* inhibits the *c-Myc*-dependent apoptosis and activates epithelial-related genes by directly binding to their promoters, resulting in the enhanced MET during reprogramming. Knockdown of *Cdh1*, an important downstream target of TFAP2C and a critical regulator of MET, antagonizes *Tfap2c*-mediated reprogramming. Our results provide a mechanistic understanding of *Tfap2c*-mediated reprogramming, which may facilitate our understanding of the molecular basis of cell identity, pluripotency, and plasticity.

## Materials and Methods

### Mice

*Oct4*-GFP transgenic allele-carrying mice (CBA/CaJ×C57BL/6 J) were purchased from the Jackson Laboratory. 129S2/SvJaeJ, C57BL/6 J and SCID mice were purchased from Charles River (Beijing, China). Animals (Specific Pathogen Free, SPF) were housed under a 12-h light/dark cycle and provided with enough food and water. Our studies met the requirement of the Care and Use of Laboratory Animals of the National Institutes of Health, and all animal studies were approved by the Ethics Committee of West China Hospital.

### Cell culture

OG2-MEFs were isolated from E13.5 mouse embryos from crossing male *Oct4*-GFP transgenic allele-carrying mice to 129S2/SvJaeJ female mice. MEFs and HEK293T cells were maintained in DMEM high-glucose media supplemented with 10% fetal bovine serum (FBS). Mouse ESCs (mESCs) and iPSCs were cultured on feeder layers or feeder free (0.1% gelatin; Sigma–Aldrich, MO, USA) with mESC medium. mESC medium consisted of KnockOut™ DMEM (Gibco, Grand Island, New York, USA) supplemented with 15% FBS (Gibco, Grand Island, New York, USA), 1 × L-glutamine (Gibco, Grand Island, New York, USA), 1 × NEAA (Gibco, Grand Island, New York, USA), 1 mM sodium pyruvate (Sigma–Aldrich, MO, USA), 1% penicillin/streptomycin (Gibco, Grand Island, New York, USA), 0.1 mM β-mercaptoethanol (Sigma–Aldrich, MO, USA), 50 mg/ml Vitamin C (Sigma–Aldrich, MO, USA), and 1000 units/ml leukemia inhibitory factor ((ESG1106, Merck, Darmstadt, Germany). All of the cell lines have been confirmed as mycoplasma contamination free with the kit from Shanghai Yise Medical Technology (PM008).

### Retrovirus production and generation of iPSCs

HEK293T cells were plated at 80–90% confluency and transfected using polyethylenimine (PEI) transfection method as previous reported^22^. The supernatant of the transfected HEK293T cells were harvested and filtered through a 0.45 μm filter (Millipore) after 24 h. Equal volumes of the four supernatants of each OSKM transcription factor with control or *Tfap2c* virus were mixed with 1 volume of fresh MEFs medium containing polybrene (Sigma–Aldrich, MO, USA) at a final concentration of 5–8 mg/ml. Two milliliter of infection mixture was used to infect 1.5 × 10^4^ OG2-MEF cells.

For iPSCs generation, 1.5 × 10^4^ OG2-MEFs at passage 2 were plated in a 12-well plate coated by 0.1% gelatin and then infected twice with retroviral supernatants. Medium was changed immediately 24 h after virus transduction and this day is termed as day 0 post-infection. Infected cells were then cultured with mESC medium post-infection and renewed daily. iPSCs colonies appeared about 6–8 days post infection. *Oct4*-GFP^+^ colonies were counted at day 12 after infection. Flow cytometry was performed to test the *Oct4*-GFP^+^ efficiency. NBT/BCIP (Roche) was used for AP staining according the instructions of the manufacturer.

### Immunofluorescence

Immunofluorescence was performed with standard procedure. The following primary antibodies were used: anti-Nanog (ab80892, abcam), anti-SSEA1 (ab16285, abcam), anti-Ki67 (ab15580, abcam), anti-α-fetoprotein (# MIA1301, Thermo Fisher Scientific, MA, USA), anti-α-smooth muscle actin (#701457, Thermo Fisher Scientific, MA, USA), anti-E-Cadherin (#14472, Cell Signaling Technology, MA, USA), anti-Epcam (ab71916, abcam), and anti-βIII tubulin (#MA1-118, Thermo Fisher Scientific, MA, USA).

### shRNAs design and vector construction

Two pairs of shRNA oligos targeting on *Tfap2c* or *Cdh1* gene were designed and constructed into PLKO.1 plasmid. The knockdown efficiency was investigated at both mRNA and protein level. The sequences of shRNA oligos are listed in supplementary Table 1.

### Quantitative RT-PCR analysis

Total mRNA was extracted with Trizol Kit (Invitrogen). 0.5 μg of total RNA was then reverse transcribed with PrimeScript™ RT reagent Kit with gDNA Eraser (Takara, Kusatsu, Japan). Quantitative RT-PCR (qRT-PCR) was performed using TB Green (Takara, Kusatsu, Japan) with a LightCycler 96^®^ machine (Roche). The primers used in the qRT-PCR assays are listed in supplementary Table 1.

### Embryonic body (EB) formation and teratoma formation

For embryonic body formation, iPSCs cells were harvested by trypsinization, plated on nonadherent bacterial culture dishes, and incubated in mESC medium without LIF. The colonies were further cultured in suspension for 3 days and then transferred onto gelatin-coated plates. After another continuous culture of 6 days, the cells were collected for later characterization. For teratoma formation 2 × 10^6^ iPSCs were injected subcutaneously into SCID mice. Tumor samples were collected within 4 weeks and processed for immunofluorescence and hematoxylin and eosin (H&E) staining following standard procedures.

### ChIP-qPCR

ChIP assays were performed using SimpleChIP^®^ Plus Enzymatic Chromatin IP Kit (Cell Signaling Technology, MA, USA) according to the manufacturer’s instructions. The primers for ChIP-qPCR are listed in supplementary Table 1.

### Cell apoptosis detection

Reprogramming cells were dissociated with TrypLE™ Express Enzyme (Gibco) to reduce damage to cells on day 4 post induction. Then cell apoptosis was detected immediately using the Annexin V-APC/PI Apoptosis Detection Kit (4 A Biotech) according to the manufacturer’s instructions. In some experimental settings, PAC-1 (Selleck, 1.25 μM) was used to induce apoptosis.

### Flow cytometry

Reprogramming cells were dissociated with TrypLE™ Express Enzyme (Gibco) at indicated time points. Cells were incubated with PE fluorescently labeled SSEA1 or Epcam monoclonal antibodies (BioLegend, CA, USA) in the dark at 4 °C for 30 min. After washed twice with PBS, the pellet was resuspended in 400 μl PBS and analyzed by the NovoCyte (Acebio).

### Immunoblotting

Immunoblotting was performed with standard procedure. The following antibodies were used in this study: anti-NANOG (#8822, Cell Signaling Technology, MA, USA), anti-TFAP2C (sc-12762, Santa Cruz Biotechnology, Santa Cruz, CA, USA), anti-GAPDH (#5174, Cell Signaling Technology, MA, USA), anti-cleaved caspase-3 (#9664, Cell Signaling Technology, MA, USA), anti-cleaved PARP1 (ab32064, abcam), anti-CDH1 (#14472, Cell Signaling Technology, MA, USA), anti-β-CATENIN (#8480, Cell Signaling Technology, MA, USA), anti-PCNA (#13110, Cell Signaling Technology, MA, USA), anti-CCND2 (#3741, Cell Signaling Technology, MA, USA), anti-CCNA2 (#91500, Cell Signaling Technology, MA, USA), anti-CCNB1 (#4135, Cell Signaling Technology, MA, USA), anti-CDK1 (#9116, Cell Signaling Technology, MA, USA), anti-CDK2 (#2546, Cell Signaling Technology, MA, USA), anti- CCND1 (#55506, Cell Signaling Technology, MA, USA), and anti-EPCAM (#93790, Cell Signaling Technology, MA, USA). Immunoblots were visualized on iBright CL1000 Imaging Systems (Thermo Fisher Scientific).

### Luciferase activity analysis

The promoter sequence of *Cdh1* was cloned into pEZX-FR01 (GeneCopoeia) before the Firefly luciferase (Fluc), the Renilla luciferase (Rluc) was used as tracer gene. mESCs were planted in 24-well plates at 2 × 10^4^ per well, and the Tfap2c or control vector (0.5 µg) and pEZX-FR01 (100 ng) were cotransfected into the cells with Lipofectamine Stem Transfection Reagent (Invitrogen, STEM00015). At 48 h after transfection, the luciferase activity was detected according to the instructions for the Luc-Pair Duo-Luciferase Assay Kit 2.0 (GeneCopoeia).

### RNA-seq

Total mRNA was extracted with Trizol Kit (Invitrogen) following the manufacturer’s protocol. RNA integrity was evaluated by using the Agilent 2100 Bioanalyzer (Agilent Technologies). The samples with RNA Integrity Number (RIN) ≥ 7 were subjected to the subsequent testing and analysis. The libraries were constructed using TruSeq Stranded mRNA LTSample Prep Kit (Illumina) according to the manufacturer’s instructions. Then these samples were sequenced on the Illumina sequencing platform (Illumina HiSeq × Ten) and 150 bp paired-end reads were generated. All of these procedures were performed by Chengdu Basebiotech Co., Ltd. And the original data of the transcription array were uploaded into SRA (SRP234686).

### Statistical analysis

For statistical analysis, mean values with ±SD. were presented in most graphs that were derived from at least three repeats of biological experiments (*N* = 3). Differences between two datasets were calculated using Unpaired two-tailed Student’s *t*-test, one-way ANOVA with Dunnett’s test, and two-way AVOVA with Sidak’s multiple comparisons test with *P* < 0.05 considered statistically significant. Statistical analysis was performed in Prism 8 (Graphpad Inc.). Sample sizes and specific tests are indicated in the figure legends. *P < 0.05, **P < 0.01, ***P < 0.001.

## Results

### *Tfap2c* is upregulated during the induction of iPSCs

To investigate the roles of *Tfap2c* in regulating reprogramming, we first examined *Tfap2c* expression levels in mouse embryonic fibroblasts (MEFs), mouse embryonic stem cells (ESCs), and iPSCs by reanalyzing a published GEO DataSets (Accession: GSE66613). *Tfap2c* was significantly higher expressed in ESCs and iPSCs compared with MEFs (Supplementary Fig. S1a). The results can be further confirmed by western blot and quantitative PCR with reverse transcription (qRT-PCR) with *Nanog* as a positive control (Supplementary Fig. S1b, c). We also noticed a gradual increase in *Tfap2c* expression during the reprogramming of MEFs with OSKM factors (Supplementary Fig. S1d). By using MEFs derived from transgenic mice carrying *Oct4*-GFP/Rosa26 (OG2-MEF), we could isolate SSEA1 positive (SSEA1^+^) and *Oct4*-GFP-positive (*Oct4*-GFP^+^) populations, which are known intermediates poised to reprogram^19,23^. We found *Tfap2c* was higher expressed in SSEA1^+^ and *Oct4*-GFP^+^ cells compared with SSEA1-negative (SSEA1^−^) and *Oct4*-GFP-negative (*Oct4*-GFP^−^) cells, respectively (Supplementary Fig. S1e, f). Previous research has demonstrated the *Tfap2c* promoter is co-occupied by OCT4, SOX2, and KLF4, indicating that cooperative binding may be responsible for the activation of *Tfap2c*^19,24^. To test this hypothesis, we examined the effect of withdrawal of individual factor from OSKM on the activation of *Tfap2c*. Indeed, withdrawal of any factors from OSKM impaired the expression of *Tfap2c* (Supplementary Fig. S1g). These results indicate that during the induction of iPSCs, *Tfap2c* is upregulated by OSKM cooperatively, and is preferentially activated in intermediates poised to reprogram, indicating a potential role of *Tfap2c* in regulating reprogramming.

### *Tfap2c* is a facilitator of somatic cell reprogramming

**Fig. 1:**
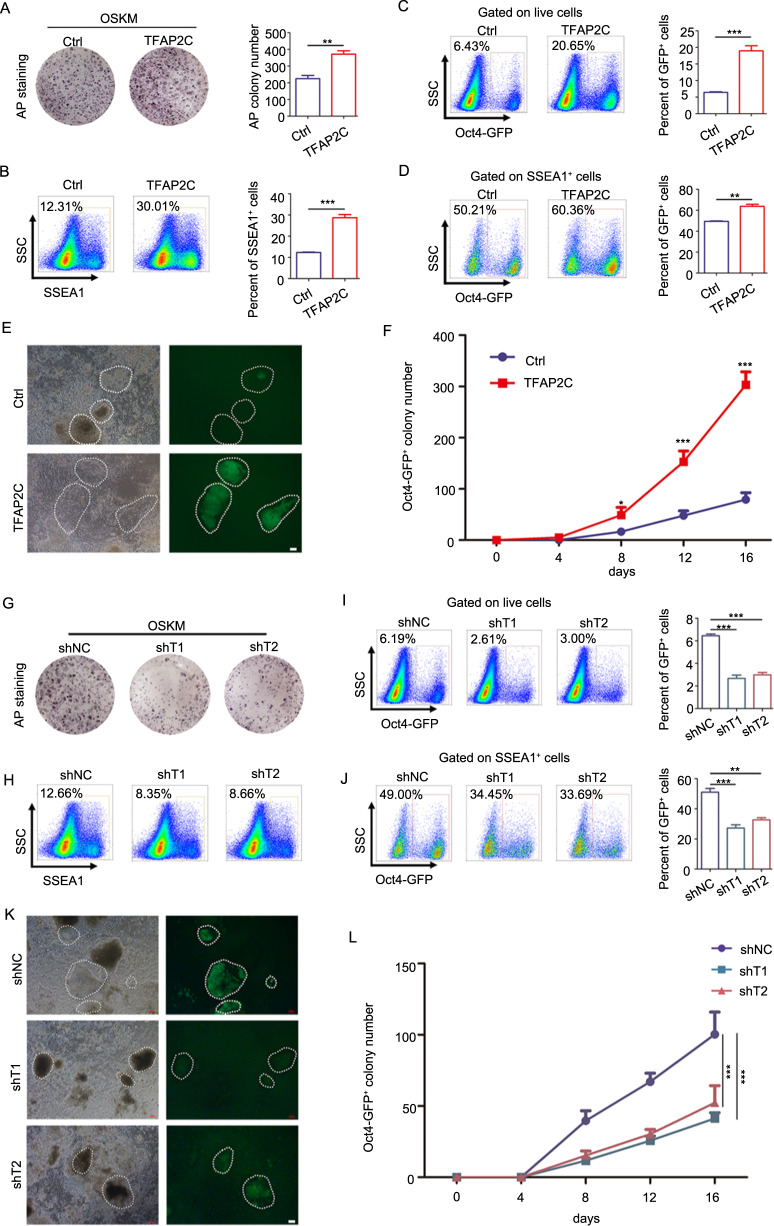
*Tfap2c* is a facilitator of somatic cell reprogramming. **a** AP-staining wells of a representative reprogramming experiment transduced with OSKM or OSKMT (day 9). **b** Flow-cytometer analysis of the SSEA1^+^ cells in reprogramming cells transduced with OSKM or OSKMT (day 9). **c** Flow-cytometer analysis of the *Oct4*-GFP^+^ cells in living cell population transduced with OSKM or OSKMT (day 12). **d** Flow-cytometer analysis of the *Oct4*-GFP^+^ cells in SSEA1^+^ cell population transduced with OSKM or OSKMT (day 12). **e** Image of *Oct4*-GFP^+^ colonies generated from MEFs transduced with OSKM or OSKMT in bright and fluorescent views (day 9). Scale bar, 100 μm. **f** Number of *Oct4*-GFP^+^ colonies of MEFs reprogrammed with OSKM and OSKMT at different time points. **g** AP-staining wells of a representative reprogramming experiment transduced with OSKM and either negative control shRNA or two shRNAs targeting *Tfap2c* (shT1 and shT2) on day 9. **h** Flow-cytometer analysis of the SSEA1^+^ cells in reprogramming cells in different groups (day 12). **i** Flow-cytometer analysis of the *Oct4*-GFP^+^ cells in living cell population in different groups (day 12). **j** Flow-cytometer analysis of the *Oct4*-GFP^+^ cells in SSEA1^+^ cell population in different groups (day 12). **k** Bright and fluorescent views of *Oct4*-GFP^+^ colonies in different groups (day 12). Scale bar, 100 μm. **l** Number of *Oct4*-GFP^+^ colonies in different groups at different time points. Significance in panels **a**, **b**, **c**, and **d** was determined by Unpaired two-tailed Student’s *t*-test. Significance in panels **f** and **l** was determined by two-way AVOVA with Sidak’s multiple comparisons test. Significance in panels **i** and **j** was determined by one-way ANOVA with Dunnett’s test. Significance summary: *P ≤ 0.05; **P ≤ 0.01; ***P ≤ 0.001. All data are presented as mean ± S.D., *N* = 3.

To investigate *Tfap2c* function in reprogramming, we transduced *Tfap2c* along with OSKM into MEFs and assessed the effect of *Tfap2c* overexpression in MEFs reprogramming (Supplementary Fig. S2a). *Tfap2c* overexpression promoted the formation of alkaline phosphatase (AP)-positive colonies (an early marker of reprogramming) and resulted in a significant increase in the fraction of SSEA1^+^ and *Oct4*-GFP^+^ (a late marker of reprogramming) intermediates compared with controls (Fig. 1a–d). Furthermore, we observed that *Tfap2c* overexpression significantly promoted the appearance of Oct4-GFP^+^ colonies (Fig. 1e, f). The OSKM+*Tfap2c* iPSCs (OSKMT-iPSCs) exhibited typical ESCs morphology, with a compact appearance and a well-defined border (Supplementary Fig. S3a). Immunofluorescence staining and qRT-PCR indicated that OSKMT-iPSCs, but not MEFs, exhibited expression of pluripotent genes at both mRNA and protein levels comparable with that of ESCs (Supplementary Fig. S3b–d). We then conducted in vitro and in vivo differentiation assays to investigate the differentiation potential of OSKMT-iPSCs. After 9 days of embryoid body (EB)-mediated in vitro differentiation (Supplementary Fig. S3e), the differentiated cells were positive for markers of three germ layers, including α-fetoprotein (endoderm marker), α-smooth muscle actin (mesoderm marker) and βIII tubulin (ectoderm marker). Teratomas also developed after subcutaneous injection of OSKMT-iPSCs into SCID mice, resulting tissues with histological structures characteristic of the three germ layers, which were further characterized by the expression of specific markers (Supplementary Fig. S3f). Thus, the OSKMT-iPSCs are functional pluripotent stem cells, as they were able to differentiate into all three germ layers in vivo and in vitro. Next, we constructed two short hairpin RNA (shRNA) vectors that showed robust downregulation of both the mRNA and protein of *Tfap2c* (Supplementary Fig. S2b). Consistent with the results of overexpression experiments, knockdown *Tfap2c* prevented the formation of iPSCs (Fig. 1g–l). Given the robust effects of *Tfap2c* in reprogramming, we speculated that *Tfap2c* may be able to substitute any of the Yamanaka factors. We found that, although *Tfap2c* could not replace *Oct4*, *Sox2*, and *Klf4*, it indeed could ‘replace’ *c-Myc* to yield comparable *Oct4*-GFP^+^ colonies (Supplementary Fig. S4a). In addition, OSKT also generated more *Oct4*-GFP^+^ colonies compared with OSK (Supplementary Fig. S4a–e). Taken together, our data indicated that *Tfap2c* is a facilitator of both OSK- and OSKM-induced somatic cell reprogramming.

### RNA-seq analysis of the effects of *Tfap2c* on somatic cell reprogramming

**Fig. 2:**
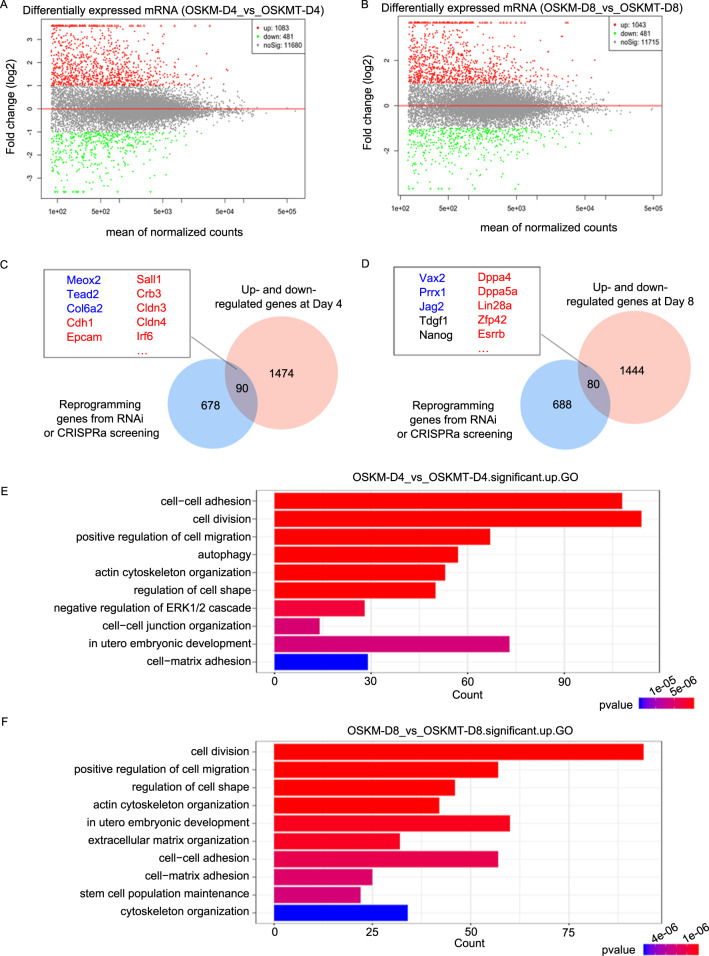
RNA-seq analysis of the effects of *Tfap2c* on somatic reprogramming. **a** Number of differentially regulated genes after *Tfap2c* overexpression compared with control on day 4. **b** Number of differentially regulated genes after *Tfap2c* overexpression compared with control on day 8. **c** Venn diagram showing the common genes between *Tfap2c*-affected genes (day 4) and the previously identified reprogramming genes. Genes that impair (or facilitate) reprogramming and downregulated (or upregulated) simultaneously in our RNA-seq dataset are highlighted in blue (or red). **d** Venn diagram showing the common genes between *Tfap2c*-affected genes (day 8) and the previously identified reprogramming genes. **e** Gene ontology analysis for genes upregulated by *Tfap2c* on day 4. **f** Gene ontology analysis for genes upregulated by *Tfap2c* on day 8.

To further confirm the effects of *Tfap2c* on reprogramming, we performed RNA-seq on day 4 and day 8 post induction. *Tfap2c* overexpression resulted in extensive transcriptional changes, and in line with the transcription activator activity of TFAP2C^25,26^, most genes were upregulated (Fig. 2a, b). By comparing *Tfap2c*-affected genes (2-fold change in expression, *P* < 0.05) with a list of ‘signature’ reprogramming genes that are dynamically regulated in reprogramming^27,28^, we found many genes (*Cdh1*, *Cldn3*, *Nanog*, and *Lin28a*) that are beneficial to reprogramming were upregulated, and genes which inhibit reprogramming were downregulated (*Mex2, Jag2*, and *Col6a2*) (Fig. 2c, d, Supplementary Table 2). Gene ontology (GO) analysis revealed that genes upregulated by *Tfap2c* are exceptionally enriched for cell–cell adhesion processes at day 4 (Fig. 2e). Other terms include those related to cell morphology, development, autophagy as well as stem cell population maintenance (Fig. 2e, f), all of which are critical events in reprogramming^29–32^. In addition, Kyoto Encyclopedia of Genes and Genomes (KEGG) analysis showed signal pathways that are essential for reprogramming, including focal adhesion, autophagy, and glycolysis were widely affected by *Tfap2c* overexpression (Supplementary Fig. S5). In conclusion, these results demonstrate that *Tfap2c* can promote reprogramming at the transcriptome level.

### *Tfap2c* exerts its effect at the beginning of the reprogramming process

**Fig. 3:**
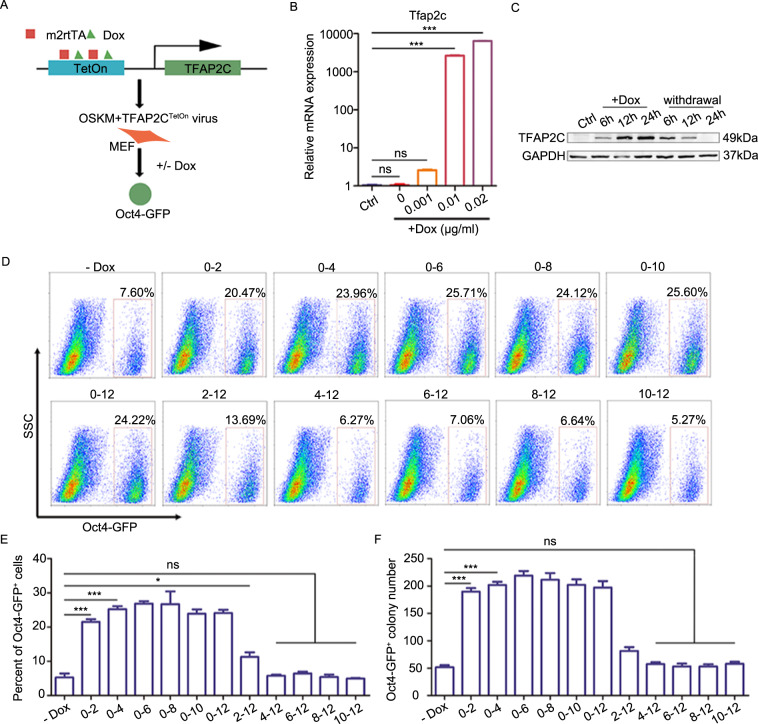
*Tfap2c* exerts its effect at the beginning of the reprogramming process. **a** Schematic depicting the strategy to determine the time window during, which *Tfap2c* can enhance somatic cell reprogramming. **b** Relative mRNA expression of *Tfap2c* in MEFs treated with Dox at different concentration. **c** Immunoblotting of TFAP2C in MEFs after treatment or withdrawal of Dox (0.02 μg/ml) at different timepoint. **d** Flow-cytometer analysis of the *Oct4*-GFP^+^ cells (day 12) in reprogramming cells after inducing *Tfap2c* expression for different durations. **e** Statistical data of *Oct4*-GFP^+^ cells (day 12) in reprogramming cells after inducing *Tfap2c* expression for different durations. **f** Number of *Oct4*-GFP^+^ colonies (day 12) in reprogramming cells after inducing *Tfap2c* expression for different durations. Significance in panels **b**, **e**, and **f** was determined by one-way ANOVA with Dunnett’s test. Significance summary: *P* > 0.05 (ns); *P ≤ 0.05; **P ≤ 0.01; ***P ≤ 0.001. All data are presented as mean ± S.D., *N* = 3.

As somatic cell reprogramming can be divided into phases with distinct molecular and phenotypic features, and factors that regulate iPSCs generation can function at different phases during reprogramming^32^, we next sought to determine the time window during which *Tfap2c* can promote somatic cell reprogramming. To this end, we developed an inducible gain-of-function approach and showed that *Tfap2c* can be induced in *Tfap2c*^TetOn^ MEFs by doxycycline (Dox) at both mRNA and protein level (Fig. 3a–c). Then, we induced *Tfap2c* expression for different durations during the somatic cell reprogramming process and examined the percentage of *Oct4*-GFP^+^ cells and the number of *Oct4*-GFP^+^ colonies at the end of reprogramming (day 12). We found that ectopic expression of *Tfap2c* in the first 2 days significantly enhanced the reprogramming efficiency (Fig. 3d–f). The efficiency could be slightly increased and reached a peak if *Tfap2c* is activated in the first 4 days, further overexpression of *Tfap2c* could not affect reprogramming significantly (Fig. 3d–f). As a complement, we also induced *Tfap2c* expression beginning at different days after the initiation of reprogramming and found that *Tfap2c* overexpression after day 4 showed no obvious effect on reprogramming (Fig. 3d–f). In conclusion, these results suggested that *Tfap2c* plays an important role in promoting iPSCs generation at the initiation phase (0–4 day) of reprogramming.

### *Tfap2c* inhibits *c-Myc*-dependent apoptosis

**Fig. 4:**
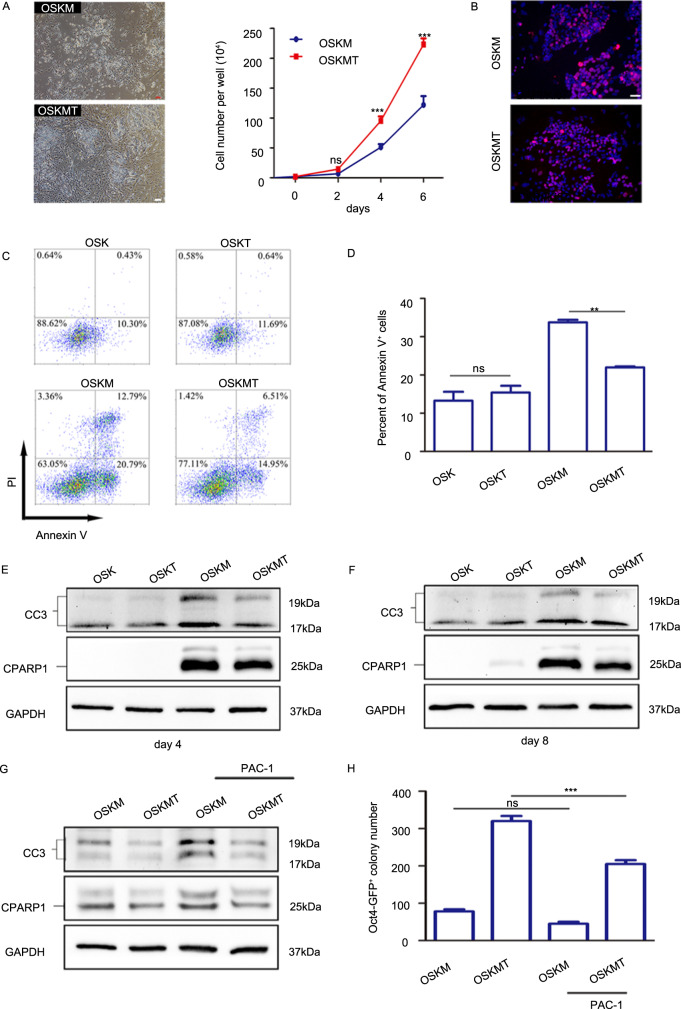
*Tfap2c* inhibits *c-Myc*-dependent apoptosis. **a** Microscopy of reprogramming cells at post-infection day 4, and the number of OSKM and OSKMT reprogramming cells at different time points was recorded. Scale bar, 100 μm. **b** Immunostaining of Ki67 in OSKM and OSKMT groups at day 4. Scale bar, 50 μm. **c** Flow-cytometer analysis of cell apoptosis after infected with OSK, OSKT, OSKM, and OSKMT on day 4. **d** The percent of Annexin V^+^ cells in OSK, OSKT, OSKM, and OSKMT group on day 4. **e** Immunoblotting of Cleaved caspase-3 (CC3) and Cleaved PARP1 (CPARP1) in MEFs after infected with OSK, OSKT, OSKM, and OSKMT on day 4. **f** Immunoblotting of CC3 and CPARP1 in MEFs after infected with OSK, OSKT, OSKM, and OSKMT on day 8. **g** Immunoblotting of CC3 and CPARP1 in MEFs after infected with OSKM and OSKMT (day 4) with or without PAC-1 (1.25 μM) treatment. **h** Number of *Oct4*-GFP^+^ colonies (day 12) in reprogramming cells treated with or without PAC-1. Significance in panels **d** was determined by one-way ANOVA with Dunnett’s test. Significance in panels **a** and **h** was determined by two-way AVOVA with Sidak’s multiple comparisons test. Significance summary: *P* > 0.05 (ns); *P ≤ 0.05; ***P ≤ 0.001. All data are presented as mean ± S.D., *N* = 3.

Given that *Tfap2c* exerts its effect at the beginning of the reprogramming, a phase which is characterized by the reduction of somatic genes, MET, inhibition of apoptosis and cellular senescence pathways^5,33–35^, we speculated *Tfap2c* may promote reprogramming by regulating these relevant events. Intriguingly, we observed a significant increased cell number upon *Tfap2c* overexpression (Fig. 4a). We then investigated the effect of *Tfap2c* on cell proliferation, cycle, and apoptosis. Although *Tfap2c* had limited effect on cell proliferation and cell cycle (Fig. 4b, Supplementary Fig. S6a), it inhibited apoptosis significantly, which was evaluated by reduction of Annexin V^+^ cells and the decreased expression of cleaved caspase-3 (CC3) and cleaved PARP1 (CPARP1) (Fig. 4c–f). Indeed, RNA-seq analysis also indicated that genes involving in positive regulation of apoptosis were downregulated, while that involving in negative regulation of apoptosis were upregulated by *Tfap2c* (Supplementary Table 3). We further demonstrated that knockdown of *Tfap2c* increased the expression of CC3 and CPARP1 in OSKM-induced reprogramming (Supplementary Fig. S6b). Interestingly, the apoptosis level was much lower in OSK-induced reprogramming compared with that in OSKM-induced reprogramming and *Tfap2c* did not change apoptosis level in OSK-induced reprogramming (Fig. 4c–f). These results are consistent with prior findings that ectopic *c-Myc* expression sensitizes cells to apoptosis^36^. To investigate whether the ability of *Tfap2c* to inhibit apoptosis requires the help of OSK, we detected the antiapoptotic effects of *Tfap2c* in the absence of OSK. Our results indicated that *c-Myc* alone could trigger apoptosis in MEFs and *Tfap2c* could inhibit apoptosis in the absence of OSK (Supplementary Fig. S6c). We further induced apoptosis by treating cells with low dose (1.25 μM) of PAC-1, a small molecule that could induce apoptosis by enhancing the enzymatic activity of procaspase-3. We found PAC-1 could promote the expression of CC3 and CPARP1 and antagonize the antiapoptotic effects of *Tfap2c* partly (Fig. 4g, h), which led to a lower reprogramming efficiency. The above data indicated *Tfap2c* can inhibit *c-Myc*-dependent apoptosis in OSKM-induced reprogramming.

### *Tfap2c* promotes MET at the initiation stage of reprogramming

**Fig. 5:**
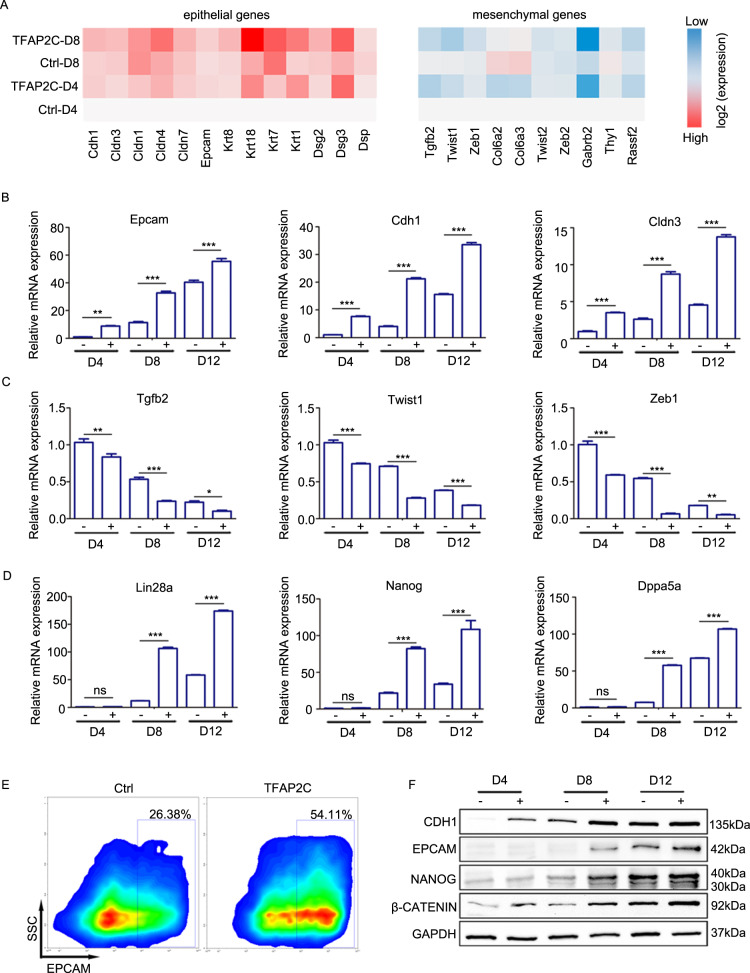
*Tfap2c* promotes MET at the initiation stage of reprogramming. **a** Heatmap of the epithelial and mesenchymal genes in reprogramming cells transduced with OSKM or OSKMT at day 4 and day 8 post induction. **b** qRT-PCR analysis of epithelial genes expression in reprogramming cells transduced with OSKM (−) or OSKMT (+) at indicated time points. **c** qRT-PCR analysis of mesenchymal genes expression in reprogramming cells transduced with OSKM or OSKMT at indicated time points. **d** qRT-PCR analysis of pluripotential genes expression in reprogramming cells transduced with OSKM or OSKMT at indicated time points. **e** Flow-cytometer analysis of the EPCAM^+^ cells in reprogramming cells transduced with OSKM or OSKMT on day 9. **f** Immunoblotting of CDH1, EPCAM, β-CATENIN, and NANOG in reprogramming cells transduced with OSKM or OSKMT at indicated time points. Significance in panels **b**, **c**, and **d** was determined by two-way AVOVA with Sidak’s multiple comparisons test. Significance summary: *P* > 0.05 (ns); *P ≤ 0.05; **P ≤ 0.01; ***P ≤ 0.001. All data are presented as mean ± S.D., *N* = 3.

The fact that *Tfap2c* promotes both OSK- and OSKM-induced reprogramming, while the apoptosis level is unaffected by *Tfap2c* in OSK-induced reprogramming prompt us to find other mechanisms that *Tfap2c* may regulate reprogramming. Another essential hallmark in the initiation phase of reprogramming is MET^9,37^. Indeed, GO and KEGG analysis revealed that genes upregulated by *Tfap2c* are exceptionally enriched for cell–cell adhesion and focal adhesion processes, both of which are key events involved in MET (Fig. 2e, f, Supplementary Fig. S5). In addition, *Tfap2c* promoted the conversion of fibroblast-like cells into epithelial-like cell at as early as day 2 (Supplementary Fig. S7). To further explore the role of *Tfap2c* on MET, we checked the expression levels of the key epithelial and mesenchymal markers in our RNA-seq dataset. *Tfap2c* overexpression leads to significant upregulation of epithelial genes, including *Cdh1*, *Epcam*, *Cldn3*, and *Krt8*. Consistent with the activation of epithelial genes, the expression level of mesenchymal regulators, such as *Twist1*, *Twist2*, *Zeb2*, *Tgfb2*, and *Zeb1*, were markedly downregulated after *Tfap2c* overexpression (Fig. 5a). To confirm this, we examined the mRNA expression level of epithelial- and mesenchymal-associated genes during reprogramming by qRT-PCR (Fig. 5b, c). Flow cytometry, western blot, and immunofluorescence analysis further validated the increased expression of epithelial regulators (Fig. 5e, f, Supplementary Fig. S8a, b). In addition, epithelial genes were downregulated after *Tfap2c* knockdown in reprogramming (Supplementary Fig. S8c). More importantly, *Tfap2c* also facilitated MET in OSK-induced reprogramming (Supplementary Fig. S9). *Tfap2c* alone could also promote MET moderately in the absence of OSKM; however, the degree was lower than that in reprogramming (Supplementary Fig. S8d). Pluripotent markers including *Nanog*, *Lin28a*, and *Dppa5a* were generally unaffected until day 8 (Fig. 5d, f). Given that *Tfap2c* exerts its effect from the beginning of reprogramming, we speculated that *Tfap2c* directly contributes to the earlier MET program, whereas its effect on the later pluripotent program is the result of an amplified cascade. Collectively, the data presented above indicated that *Tfap2c* facilitates MET, which may in turn activate downstream genes including pluripotent genes.

### *Tfap2c* activates MET regulators directly

**Fig. 6:**
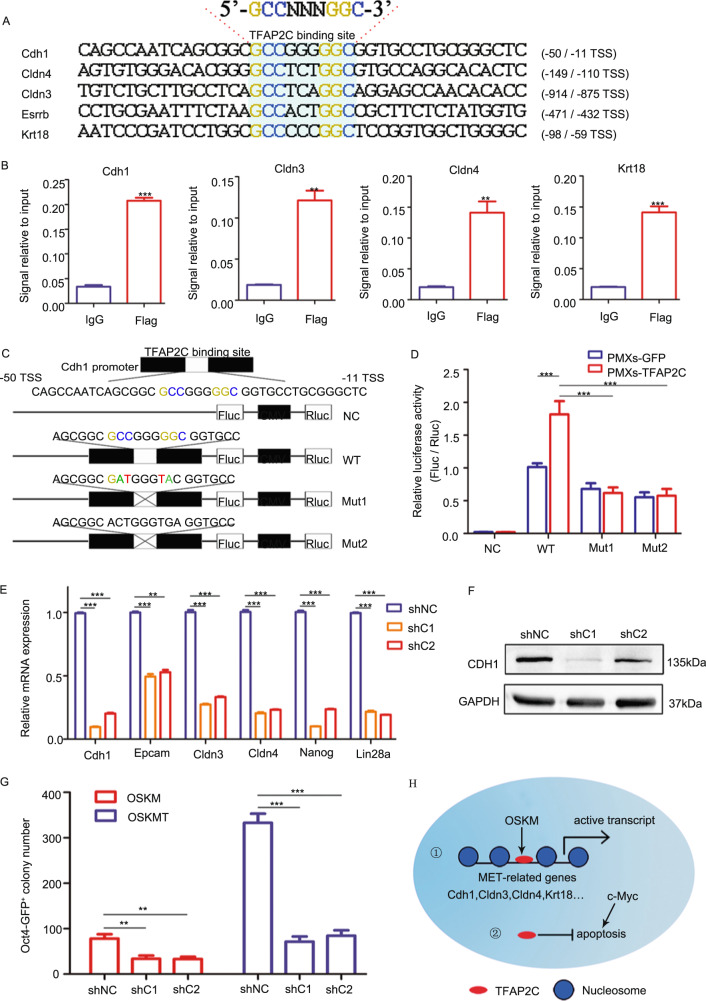
*Tfap2c* activates MET regulators directly. **a** Presence of *Tfap2c*-binding sequence in the promoter of the indicated genes. **b** The binding of Flag-tagged *Tfap2c* on the *Cdh1*, *Cldn3*, *Cldn4*, and *Krt18* loci was determined by ChIP-qPCR. Rabbit IgG was used as a control. **c** Schematic representation the reporter designed for determining *Tfap2c* binding on *Cdh1* loci. NC, negative control. **d** *Tfap2c* enhanced the wild type (WT) *Cdh1* reporter but not the mutated (Mut) ones in mESCs. Luciferase activity was detected at 36 h post-transfection. NC negative control. **e** qRT-PCR analysis for the MET genes using RNA lysates from MEFs reprogrammed with OSKMT plus control shRNA or two shRNAs for *Cdh1* (shC1 and shC2) on day 4. **f** Western blot to test *Cdh1*-knockdown efficiency in mESCs. **g** Number of *Oct4*-GFP^+^ colonies (day 12) in different groups. **h** A proposed working model schematically representing the main message of our work. Significance in panels **b** was determined by Unpaired two-tailed Student’s *t*-test. Significance in panels **d** was determined by two-way AVOVA with Sidak’s multiple comparisons test. Significance in panels **e** and **g** was determined by one-way ANOVA with Dunnett’s test. Significance summary: **P ≤ 0.01; ***P ≤ 0.001. All data are presented as mean ± S.D., *N* = 3.

To figure out how *Tfap2c* regulates MET, we performed chromatin immunoprecipitation (ChIP)-qPCR given that canonical TFAP2C-binding motif GCCNNNGGC exist in the promoter regions of many MET genes (Fig. 6a). Our results demonstrated that the genomic loci of these MET genes were occupied by TFAP2C (Fig. 6b), indicating TFAP2C may activate MET by binding the promoter directly. Among many of the TFAP2C targets and MET regulators, we selected *Cdh1* as an important candidate, which may meditate *Tfap2c*-induced reprogramming for its multifunction roles in cell–cell adhesion and maintenance of pluripotency^38–42^. We then mutated the canonical motif of TFAP2C in the *Cdh1* promoter and showed that it blocked the activation effects of TFAP2C in a reporter assay (Fig. 6c, d). Next, we constructed two shRNA vectors that showed robust downregulation of both the mRNA and protein of Cdh1 (Fig. 6e, f, Supplementary Fig. S10a). Indeed, knockdown of *Cdh1* impaired the expression of epithelial and pluripotent genes (Fig. 6e) and led to a blocked MET (Supplementary Fig. S10b) and pluripotent program. More importantly, the number of AP^+^ and *Oct4*-GFP^+^ colonies were decreased remarkably upon inhibition of *Cdh1* (Fig. 6g, Supplementary Fig. S10c, d). Collectively, our results demonstrated that *Tfap2c* promotes reprogramming by regulating MET directly.

## Discussion

In this study, we report that the transcription factor *Tfap2c* acts as a facilitator to somatic cell reprogramming. Mechanistically, *Tfap2c* inhibits the *c-Myc*-dependent cell apoptosis and activates epithelial-related genes by binding to the promoter directly, resulting in the enhanced MET and reprogramming efficiency (Fig. 6h).

Since the first report of iPSCs, the molecular mechanisms of reprogramming have been extensively investigated. A better mechanistic understanding of reprogramming is essential to iPSCs biology and improvement of the efficiency and quality of iPSCs for therapeutic use. Typically, introduction of factors that are highly expressed in ESCs, such as *Lin28a* and *Nanog*, while inhibition of genes that show low expression in ESCs, such as *c-Jun* and *p53*, augments reprogramming^43–45^. In our study, *Tfap2c* was significantly higher expressed in ESCs and iPSCs compared with MEFs and was preferentially activated in intermediates poised to reprogram, indicating a potential role of *Tfap2c* in promoting somatic cell reprogramming. In addition, we found OSKT could generate comparable *Oct4-GFP*^+^ colonies compared with OSKM, which may avoid undesirable genetic modification in reprogramming caused by oncogenes *c-Myc*^46^. Collectively, our results highlight the important potential of *Tfap2c* regarding the efficiency and safety in reprogramming, which are key issues for the further application of iPSCs.

Mechanistic analyses have demonstrated that the first step of reprogramming is “initiation”, and it is characterized by loss of somatic cell gene expression, changes of metabolism and cytoskeleton organization, increase in cellular proliferation, inhibition of apoptosis and initiation of MET^47–49^. Given that *Tfap2c* exerts its effects at the initiation phase of the reprogramming, we speculated *Tfap2c* may promote reprogramming by regulating relevant events involving in this phase. Cell apoptosis has been identified as a barrier to reprogramming^35,50,51^, one of the well-characterized mechanisms is the activation of *p53* pathway and the accompanying cell senescence and apoptosis^33,50^. In addition, although *c-Myc* enhances the overall reprogramming efficiency, *c-Myc* can also trigger cell apoptosis via *p53*-dependent and -independent way, which may limit reprogramming^35,50^. This seems counterintuitive since exogenous *c-Myc* has traditionally been considered as a facilitator for reprogramming. However, recent studies had revealed dual role of *c-Myc* in reprogramming, and indicated *c-Myc* can negatively influences reprogramming by inducing cell apoptosis and creating an epigenetic barrier together with NCoR/SMRT corepressors^52^. In our present study, we found *Tfap2c* can attenuated the ‘side effect’ of *c-Myc* in reprogramming by inhibiting *c-Myc*-induced apoptosis, in agreement with previous results that *Tfap2c* decreased apoptosis in an activated *Neu* model of mammary carcinogenesis^53^. Although the precise mechanism is remained to further investigated, the fact that AP2 transcription factor family function as a negative regulator of *c-Myc* by impairing DNA binding of *c-Myc* at specific sites prompts us to speculate *Tfap2c* may inhibit *c-Myc*-dependent apoptosis in similar way^54^.

Accumulating evidence indicates that MET is indispensable during early somatic cell reprogramming in mouse embryonic fibroblasts and human fibroblasts, and that blocking this step can immensely impair reprogramming efficiency^39^. During MET, mesenchymal cells progressively undergo the establishment of apicobasal polarity, which accompanied by formation of tight junctions and the reorganization of cytoskeletal structures^9^. It has been reported that *Tfap2c* played important role in the identity of a wide variety of ectodermal and endodermal-derived epithelia, including mammary, colon, and skin epidermis^55–58^. For example, loss of *Tfap2c* in luminal breast cancer cells induced a luminal to basal cell transition and was associated with the development of a mesenchymal expression pattern^42^. In our present study, we found genes upregulated by *Tfap2c* are exceptionally enriched for cell–cell adhesion processes, cell morphology, cytoskeleton organization as well as cell migration. In addition, *Tfap2c* overexpression lead to significant upregulation of epithelial genes by binding to the promoter directly. Given the potential of TFAP2C in regulating *Cdh1* and the indispensable role of *Cdh1* in MET, somatic cell reprogramming and maintenance of pluripotency^38,39,41,42^, we selected *Cdh1* as an important candidate, which may meditate *Tfap2c*-induced reprogramming. Our data demonstrated that downregulation of the expression of *Cdh1* significantly antagonized the effect of *Tfap2c*-meditated reprogramming, further emphasizing the critical role of MET in reprogramming.

*Tfap2c* is known for its central role in trophoblast development and in the conversion of mouse fibroblasts into functional-induced trophoblast stem-like cells (iTSCs)^14,17^. Previous and our present studies also demonstrate the importance of *Tfap2c* in somatic cell reprogramming^19,23^. More importantly, a combination of five transcription factors including *Tfap2c* (*Gata3*, *Tfap2c*, *Eomes*, c-*Myc*, and *Esrrb*) can reprogram mouse fibroblasts into both iPSCs and iTSCs^20^. These results agree with the idea that iTSCs and iPSCs may share common transcriptional networks to establish self-renewal and may help us to better understand the internal connection between them^16^. Indeed, generation of iPSCs and iTSCs both required the activation of MET in early stage. Given the significant role of *Tfap2c* in epithelial cell identity, we speculated that *Tfap2c*-induced MET may be a generic mechanism in *Tfap2c*-induced reprogramming toward epithelial cells. Collectively, our results emphasize the importance of *Tfap2c* in regulating cell apoptosis and MET during somatic cell reprogramming and therefore provide better understanding of reprogramming mechanisms.

## Supplementary information

Supplementary Figure Legends

Supplementary Figure 1

Supplementary Figure 2

Supplementary Figure 3

Supplementary Figure 4

Supplementary Figure 5

Supplementary Figure 6

Supplementary Figure 7

Supplementary Figure 8

Supplementary Figure 9

Supplementary Figure 10

supplementary table legends

Supplementary Table 1

Supplementary Table 2

Supplementary Table 3

## Data Availability

All the data needed to evaluate the conclusions in the paper are present in the paper or in the Supplementary Materials.
