# Supplementary material for: TFAP2C facilitates somatic cell reprogramming by inhibiting c-Myc-dependent apoptosis and promoting mesenchymal-to-epithelial transition
Source: Cell Death Dis. 2020 Jun 25;11(6):482. doi: 10.1038/s41419-020-2684-9 (PMC7316975; doi:10.1038/s41419-020-2684-9)
Supplement: Supplementary file 1 — Supplementary Figure Legends [file 41419_2020_2684_MOESM1_ESM.docx]

**Supplementary Figure S1. *Tfap2c* is upregulated during the induction of iPSCs**

1. The heat maps of top 50 differentially expressed mRNA in MEFs, iPSCs and ESCs.
2. Relative mRNA expression of *Tfap2c* and *Nanog* in MEFs, iPSCs and ESCs.
3. Immunoblotting of NANOG and TFAP2C in MEFs, iPSCs and ESCs.
4. Relative mRNA expression of *Tfap2c* and *Nanog* during reprogramming at the indicated days.
5. Relative mRNA expression of *Tfap2c* in MEFs, SSEA1^－^ and SSEA1^+^ cells at day 8.
6. Relative mRNA expression of *Tfap2c* in MEFs, *Oct4*-GFP^－^and *Oct4*-GFP^+^ cells at day 12.
7. Relative mRNA expression of *Tfap2c* after withdrawal of individual factors from OSKM (day4).

Significance in panels (b), (e), (f) and (g) was determined by one-way ANOVA with Dunnett’s test. Significance summary: ***P* ≤ 0.01; ****P* ≤ 0.001. All data are presented as mean ± S.D., N = 3.

**Supplementary Figure S2. Successful overexpression and knockdown of *Tfap2c.***

1. qRT-PCR and western blot to test *Tfap2c* overexpression in MEFs.
2. qRT-PCR and western blot to test *Tfap2c* knockdown efficiency in mESCs.

Significance in panels (a) was determined by Unpaired two-tailed Student’s t-test. Significance in panels (b) was determined by one-way ANOVA with Dunnett’s test. Significance summary: ****P* ≤ 0.001. All data are presented as mean ± S.D., N = 3.

**Supplementary Figure S3. The OSKMT-iPSCs are functional pluripotent stem cells.**

1. Morphology of OSKMT iPSC line. Scale bar, 100μm.
2. Immunostaining of pluripotent markers SSEA1 (red) and Nanog (red) in OSKMT iPSC lines. Nuclear staining by DAPI (blue). Scale bar, 50μm.
3. qRT-PCR analysis shows epithelial gene expression in MEFs, OSKMT iPSCs and ESCs. Relative mRNA expression was represented relative to expression in MEFs.
4. qRT-PCR analysis shows pluripotent gene expression in MEFs, OSKMT iPSCs and ESCs. Relative mRNA expression was represented relative to expression in MEFs.
5. Immunostaining confirming *in vitro* differentiation into all three germ layers. Scale bar, 50μm.
6. H&E staining of teratoma generated from OSKMT iPSCs showing representative ectodermal, mesodermal and endodermal tissues (upper). Immunostaining confirming *in vivo* differentiation into all three germ layers (lower). Scale bar, 50μm.

Significance in panels (c) and (d) was determined by one-way ANOVA with Dunnett’s test. Significance summary: ****P* ≤ 0.001. All data are presented as mean ± S.D., N = 3.

**Supplementary Figure S4. *Tfap2c* facilitates OSK-induced reprogramming.**

1. Number of *Oct4*-GFP^+^ colonies (day 16) in reprogramming cells transduced with indicated factors.
2. AP-stained wells of a representative reprogramming experiment transduced with OSK or OSKT on day 12.
3. Flow cytometer analysis of the *Oct4*-GFP^+^ cells in living cell population transduced with OSK or OSKT on day 16. Scale bar, 100μm.
4. Image of *Oct4*-GFP^+^ colonies generated from MEFs transduced with OSK or OSKT in fluorescent views on day 16.
5. Number of *Oct4*-GFP^+^ colonies of MEFs reprogrammed with OSK and OSKT at different time points.

Significance in panels (b) and (c) was determined by Unpaired two-tailed Student’s t-test. Significance in panels (a) and (e) was determined by two-way AVOVA with Sidak’s multiple comparisons test. Significance summary: **P* ≤ 0.05; ***P* ≤ 0.01; ****P* ≤ 0.001. All data are presented as mean ± S.D., N = 3.

**Supplementary Figure S5. KEGG analysis for genes upregulated by *Tfap2c* on day 4 and day 8.**

1. KEGG analysis for genes upregulated by *Tfap2c* on day 4.
2. KEGG analysis for genes upregulated by *Tfap2c* on day 8.

**Supplementary Figure S6. *Tfap2c* inhibits *c-Myc*-dependent apoptosis**

1. Immunoblotting of markers related to cell proliferation and cell cycle at day 4 in OSKM and OSKMT groups.
2. Immunoblotting of CC3 and CPARP1 in reprogramming cells after transduced with OSKM and either negative control shRNA (shNC) or two shRNAs targeting *Tfap2c* (shT1 and shT2) on day 4.
3. Immunoblotting of CC3 and CPARP1 in MEFs after transduced with indicated factors.

**Supplementary Figure S7. *Tfap2c* promoted the conversion of fibroblast-like cells into epithelial-like cells.**

1. Microscopy of reprogramming cells at post-infection day 2. Images show that *Tfap2c* promotes MET in reprogramming. Scale bars, 100 µm. All data are presented as mean ± S.D., N = 3.

**Supplementary Figure S8. *Tfap2c* promotes MET in cell reprogramming**

1. Immunostaining of CDH1 in OSKM and OSKMT groups at day 4. Scale bar, 50μm.
2. Immunostaining of EPCAM in OSKM and OSKMT groups at day 4. Scale bar, 50μm.
3. qRT-PCR analysis of epithelial genes expression in reprogramming cells transduced with OSKM and either negative control shRNA (shNC) or two shRNAs targeting Tfap2c (shT1 and shT2) on day 4.
4. qRT-PCR analysis of epithelial and mesenchymal genes expression after transduced with *Tfap2c* alone in MEFs.

Significance in panels (c) was determined by one-way ANOVA with Dunnett’s test. Significance in panels (d) was determined by Unpaired two-tailed Student’s t-test.

Significance summary: *P* > 0.05 (ns); **P* ≤ 0.05; ***P* ≤ 0.01; ****P* ≤ 0.001. All data are presented as mean ± S.D., N = 3.

**Supplementary Figure S9. *Tfap2c* promotes MET in OSK-induced reprogramming**

1. qRT-PCR analysis of epithelial genes expression in reprogramming cells transduced with OSK or OSKT at indicated time points.
2. qRT-PCR analysis of pluripotent genes expression in reprogramming cells transduced with OSK or OSKT at indicated time points.

Significance in panels (a) and (b) was determined by two-way AVOVA with Sidak’s multiple comparisons test. Significance summary: *P* > 0.05 (ns); **P* ≤ 0.05; ***P* ≤ 0.01; ****P* ≤ 0.001.

**Supplementary Figure S10. Knockdown of *Cdh1* impairs *Tfap2c*-induced reprogramming.**

1. Immunostaining to test *Cdh1* knockdown efficiency in mESCs. Scale bars, 50 µm.
2. Microscopy of reprogramming cells at post-infection day 6. Images show that knockdown *Cdh*1 impairs MET in OKSM and OSKMT induced reprogramming. Scale bars, 100 µm.
3. AP-stained wells of a representative reprogramming experiment transduced with OSKM and OSKMT with shNC or two shRNAs targeting *Cdh1* on day 12.
4. Image of *Oct4*-GFP^+^ colonies generated from MEFs transduced with OSKM and OSKMT with shNC or two shRNAs targeting *Cdh1* in fluorescent views on day 12. Scale bar, 100μm.
