# Supplementary figures and images for: TFAP2C facilitates somatic cell reprogramming by inhibiting c-Myc-dependent apoptosis and promoting mesenchymal-to-epithelial transition
Source: Cell Death Dis. 2020 Jun 25;11(6):482. doi: 10.1038/s41419-020-2684-9 (PMC7316975; doi:10.1038/s41419-020-2684-9)

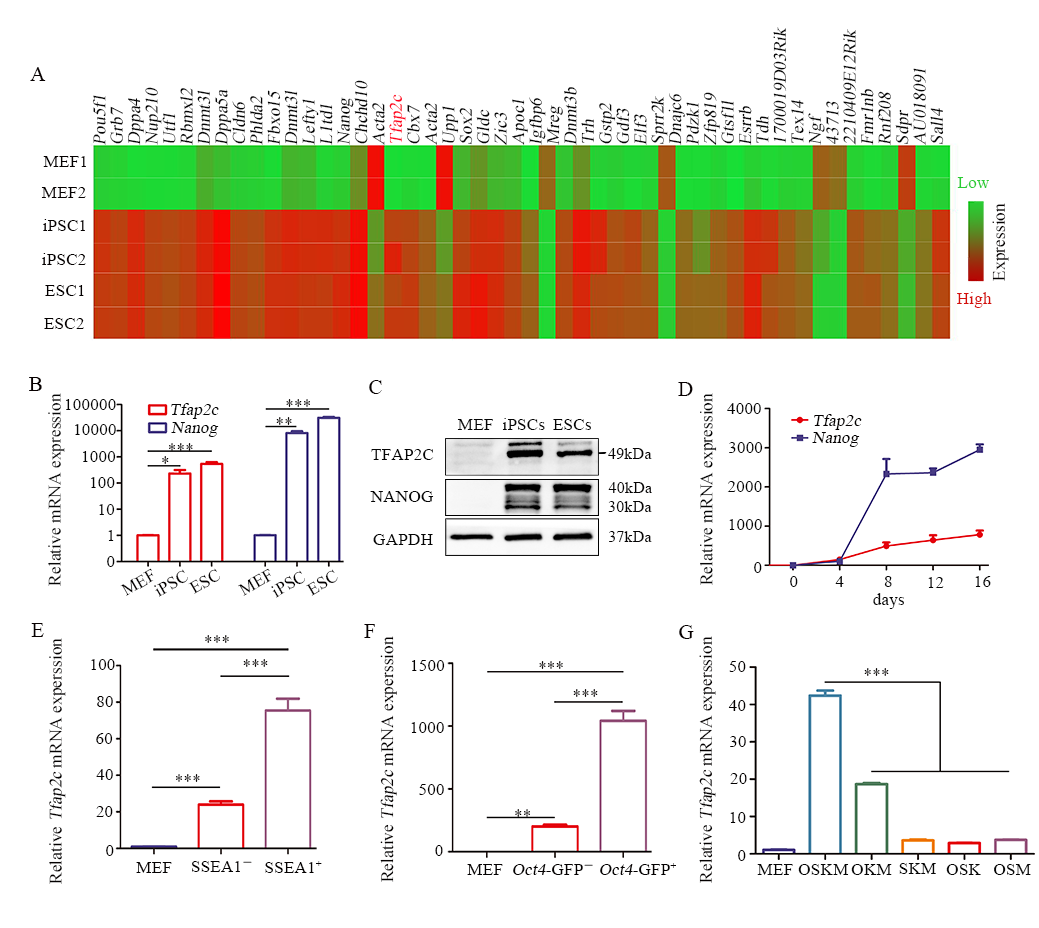

Supplement: Supplementary file 2 — Supplementary Figure 1 [file 41419_2020_2684_MOESM2_ESM.tif]

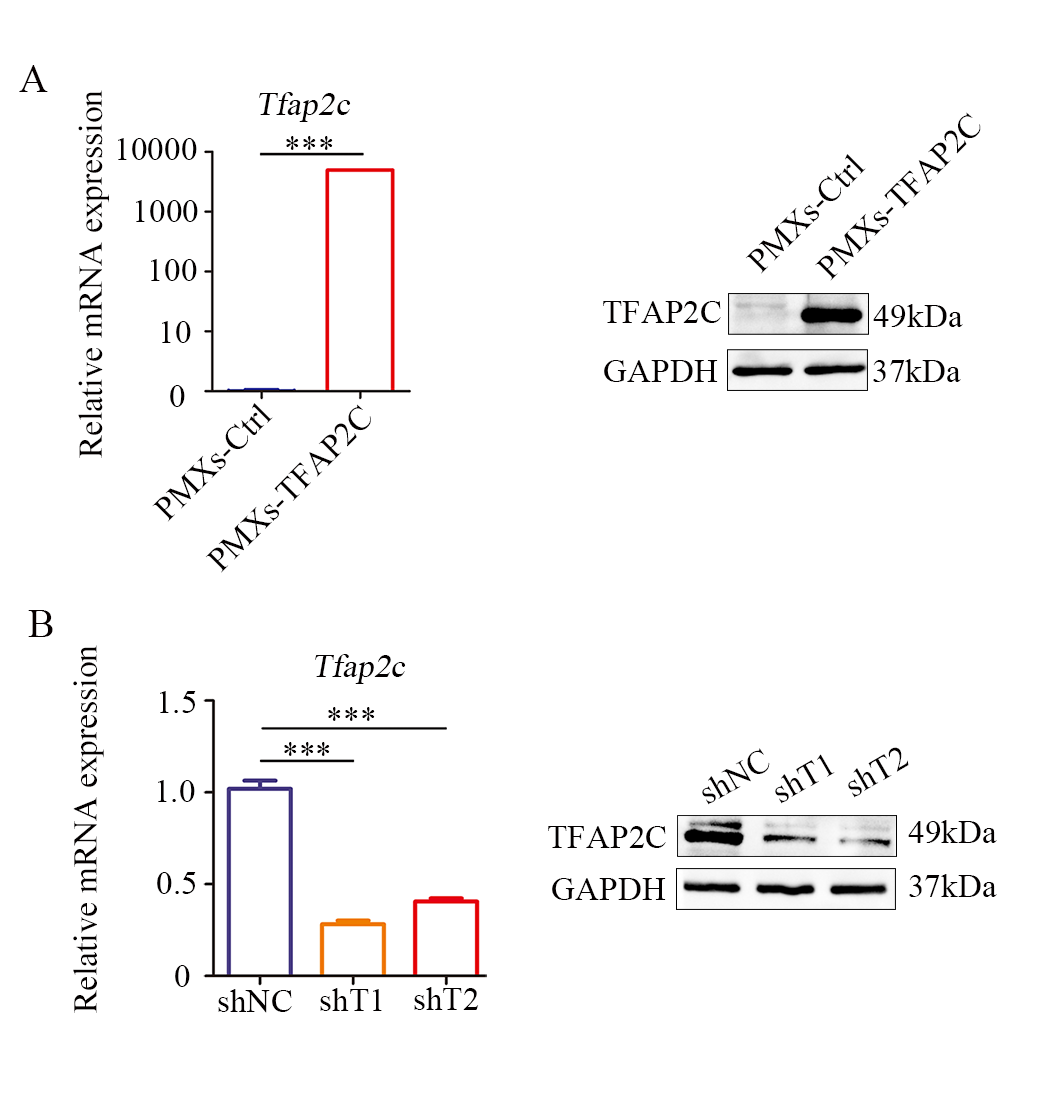

Supplement: Supplementary file 3 — Supplementary Figure 2 [file 41419_2020_2684_MOESM3_ESM.tif]

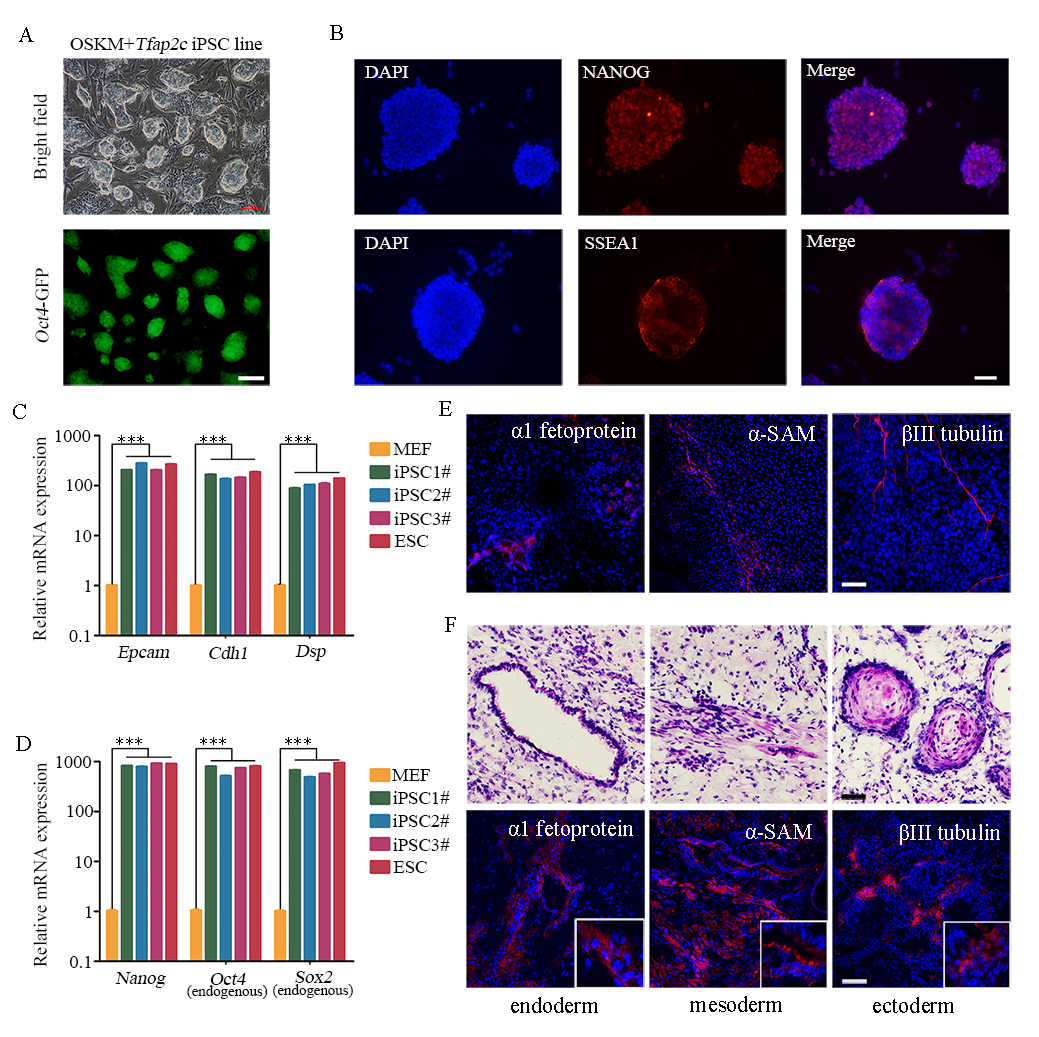

Supplement: Supplementary file 4 — Supplementary Figure 3 [file 41419_2020_2684_MOESM4_ESM.tif]

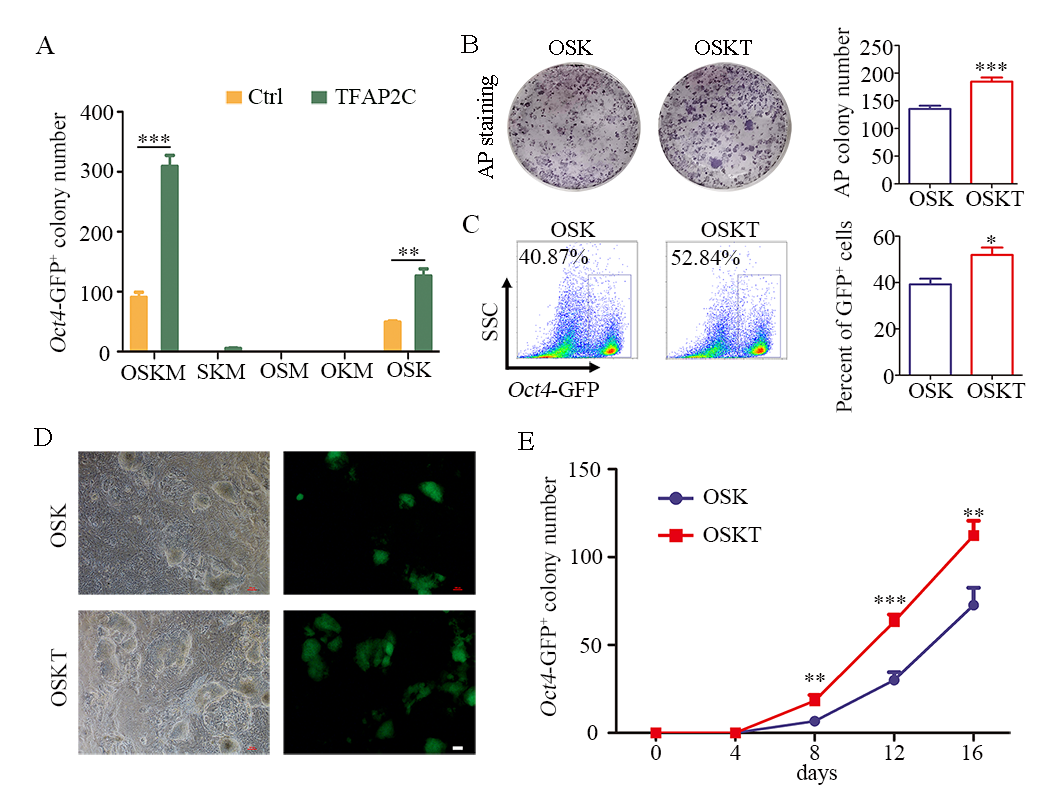

Supplement: Supplementary file 5 — Supplementary Figure 4 [file 41419_2020_2684_MOESM5_ESM.tif]

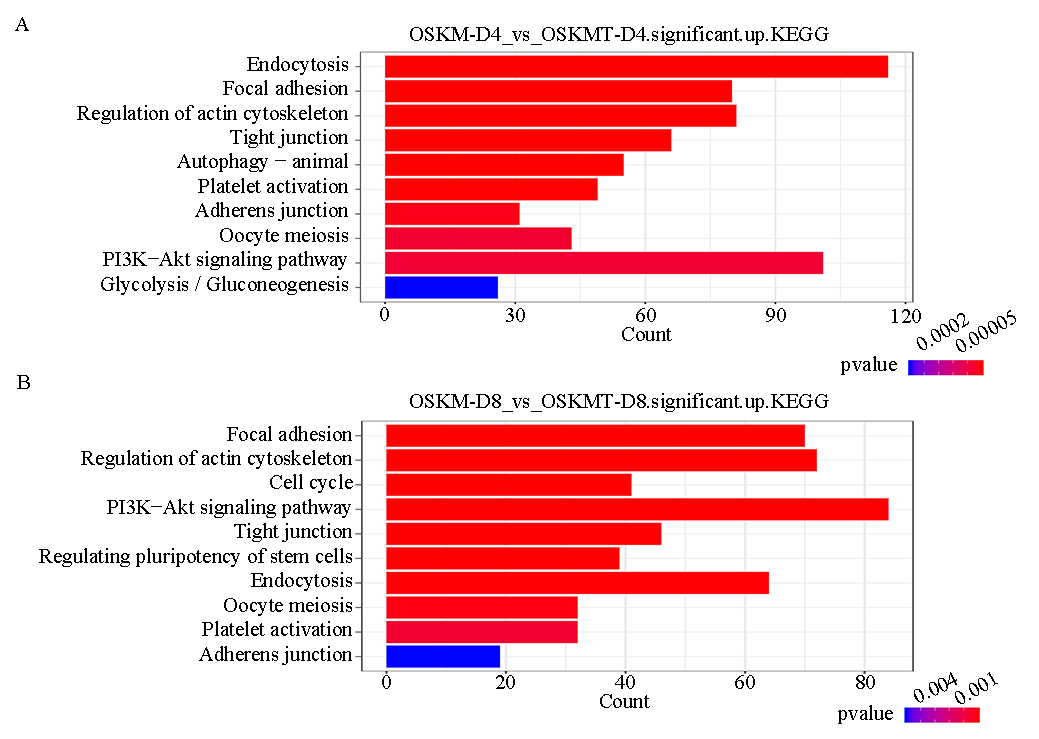

Supplement: Supplementary file 6 — Supplementary Figure 5 [file 41419_2020_2684_MOESM6_ESM.tif]

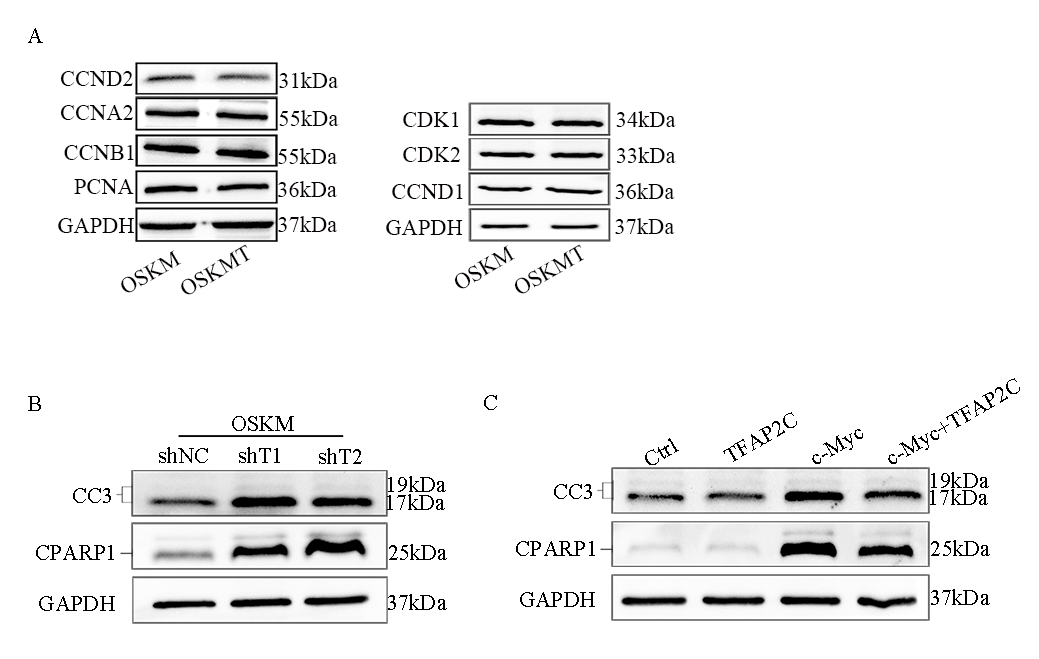

Supplement: Supplementary file 7 — Supplementary Figure 6 [file 41419_2020_2684_MOESM7_ESM.tif]

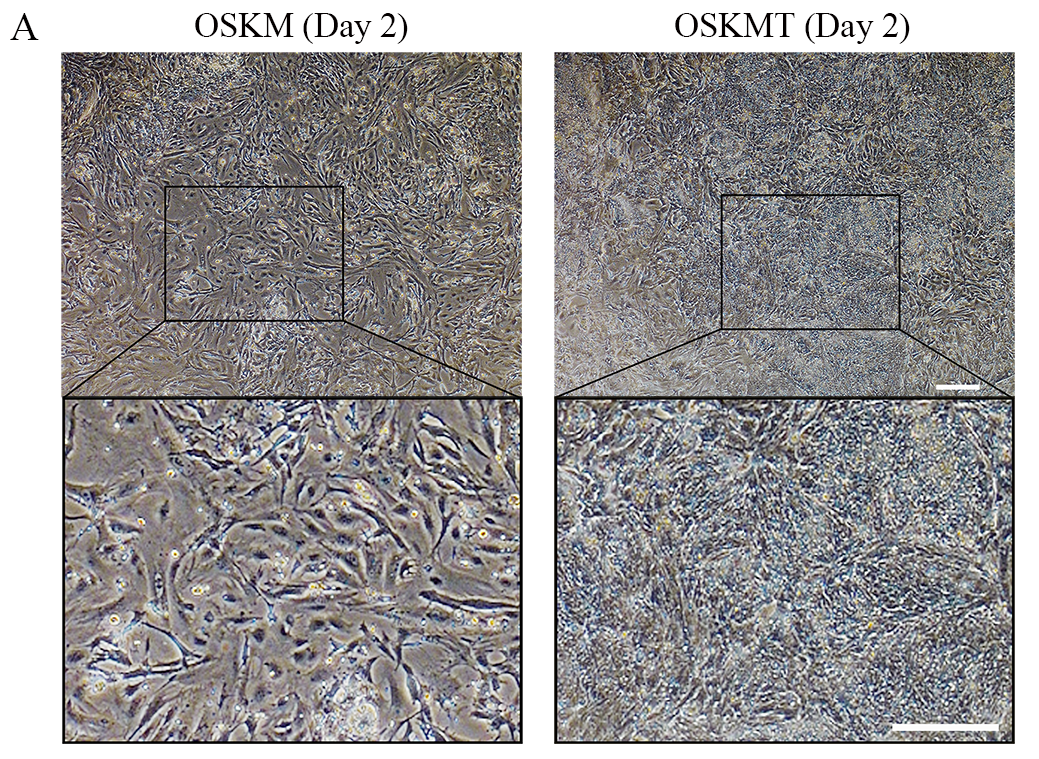

Supplement: Supplementary file 8 — Supplementary Figure 7 [file 41419_2020_2684_MOESM8_ESM.tif]

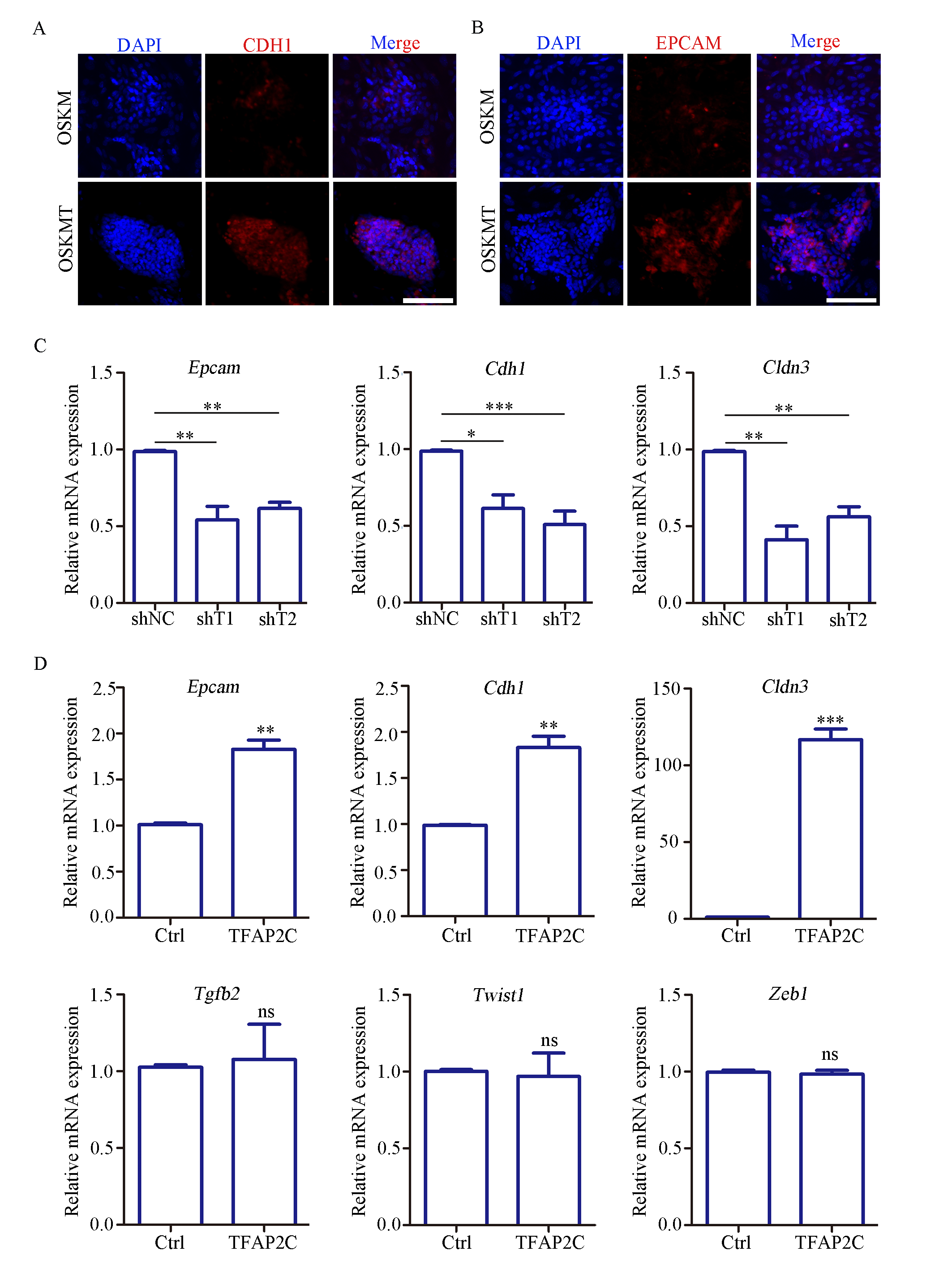

Supplement: Supplementary file 9 — Supplementary Figure 8 [file 41419_2020_2684_MOESM9_ESM.tif]

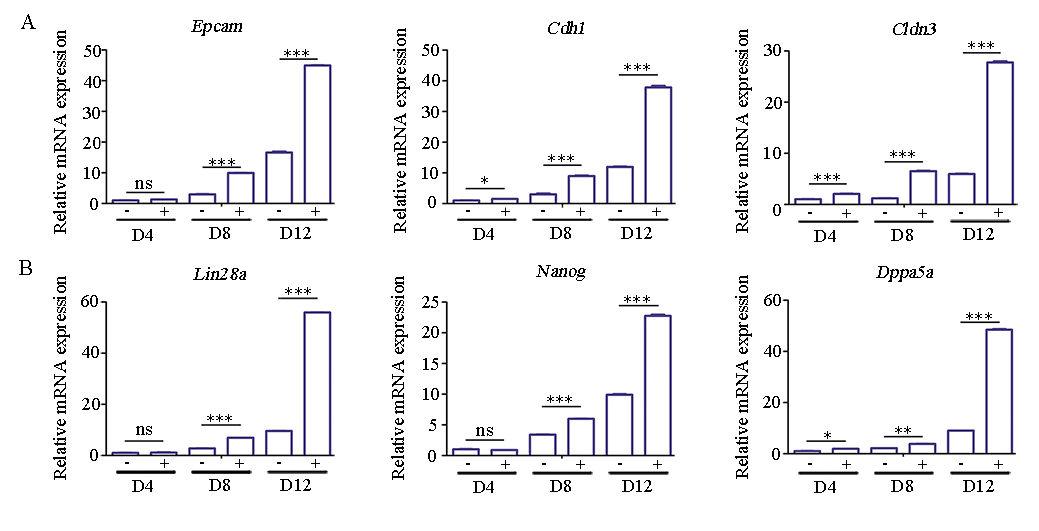

Supplement: Supplementary file 10 — Supplementary Figure 9 [file 41419_2020_2684_MOESM10_ESM.tif]

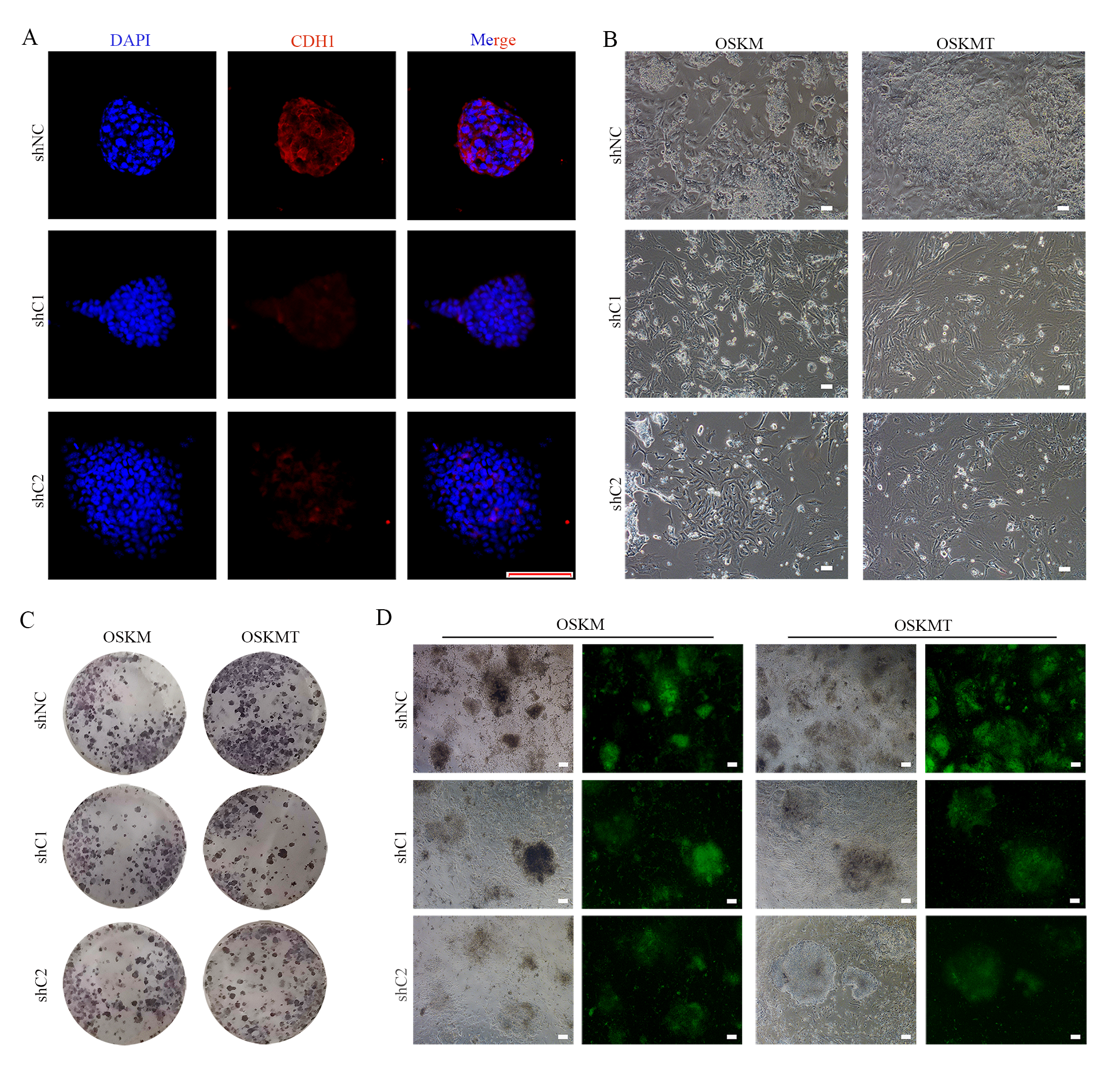

Supplement: Supplementary file 11 — Supplementary Figure 10 [file 41419_2020_2684_MOESM11_ESM.tif]
