# Supplementary material for: TFAP2C facilitates somatic cell reprogramming by inhibiting c-Myc-dependent apoptosis and promoting mesenchymal-to-epithelial transition
Source: Cell Death Dis. 2020 Jun 25;11(6):482. doi: 10.1038/s41419-020-2684-9 (PMC7316975; doi:10.1038/s41419-020-2684-9)
Supplement: Supplementary file 12 — supplementary table legends [file 41419_2020_2684_MOESM12_ESM.docx]

**Supplementary Table 1. Sequences of primers and shRNAs used in this study**

**Supplementary Table 2. Comparison of Tfap2c-affected genes with a list of ‘signature’ reprogramming genes**

**Supplementary Table 3. Tfap2c affects the expression of apoptosis-related genes in reprogramming**
