# Supplementary material for: TFAP2C facilitates somatic cell reprogramming by inhibiting c-Myc-dependent apoptosis and promoting mesenchymal-to-epithelial transition
Source: Cell Death Dis. 2020 Jun 25;11(6):482. doi: 10.1038/s41419-020-2684-9 (PMC7316975; doi:10.1038/s41419-020-2684-9)
Supplement: Supplementary file 13 — Supplementary Table 1 [file 41419_2020_2684_MOESM13_ESM.docx]

| **Primers used for qRT-PCR** | | |
| --- | --- | --- |
| **Gene** | **Direction** | **Sequence (5'→3')** |
| Epcam | F | AAGAACCGACAAGGACACGG |
|  | R | TCTGATGGTCGTAGGGGCTT |
| Cdh1 | F | CGATTACGAGGGCAGTGGTT |
|  | R | TGTCCGCCAGCTTCTTGAAT |
| Cldn3 | F | GTACAAGACGAGACGGCCAA |
|  | R | CGTACAACCCAGCTCCCATC |
| Tfap2c | F | TGAAGAGGATTGCGAGGATCG |
|  | R | AGATGCGAGTAATGGTCGGC |
| Nanog | F | TACCTCAGCCTCCAGCAGAT |
|  | R | CACTGGTTTTTCTGCCACCG |
| Dppa5a | F | TGACCCTCGTGACCCGTAAA |
|  | R | CATTCGAGATCCCTGTGGGC |
| Zeb1 | F | GGGACCTCAATGCACTTCCA |
|  | R | GTGGCTGACTGGGAGACAAA |
| Twist1 | F | CAGAGATTCCCAGAGGGGCA |
|  | R | TCGTCAAAAAGTGGGGTGGG |
| Tgfb2 | F | TCCCCTCCGAAAATGCCATC |
|  | R | ACTCTGCCTTCACCAGATTCG |
| Lin28a | F | TCGGTGTCCAACCAGCAGTT |
|  | R | GGCGGTCATAGACAGGAAGC |
| Oct3/4 (endogenous) | F | GGGCTCTCCCATGCATTCAA |
|  | R | CCCAAAGCTCCAGGTTCTCTT |
| Sox2 (endogenous) | F | GCCCAGTAGACTGCACATGG |
|  | R | TTTGCACCCCTCCCAATTCC |
| Dsp | F | GCAGAAGGAAGACGATTCCAAGA |
|  | R | TTCTGCATGGTTTCCGAGCC |
| β-actin | F | ATCGCTGCGCTGGTCG |
|  | R | CCACGATGGAGGGGAATACAG |
| **Primers used for ChIP-qPCR** | | |
| Cdh1 | F | CCCTGCAGTTCCTTGGCT |
|  | R | CTCCCACACCAGTGAGCAG |
| Cldn3 | F | TGGGACTACAGGCATGGCA |
|  | R | CGTCTAGCCAGGTGTGTTGG |
| Cldn4 | F | GGATGAGTGTGGGACACGG |
|  | R | GGGTTCACAGGAGACCTTGAT |
| krt18 | F | GTTATCCACTCCCTGTGTCGG |
|  | R | TGGCAGTCCTCGACCTGTTG |
|  | | |
|  | | |
| **shRNA sequences** | | |
| **cohesive end (CCGG)-Sense-Loop-Antisense-terminator (TTTTTG)** | | |
| Negative control (NC) | F | CCGGCAACAAGATGAAGAGCACCAACTCGAG TTGGTGCTCTTCATCTTGTTGTTTTTG |
|  | R | AATTCAAAAACAACAAGATGAAGAGCACCAACTCGAGTTGGTGCTCTTCATCTTGTTG |
| mouse Tfap2c (shT1) | F | CCGGAGCCGCTCTGCAAGTCTAATACTCGAGTATTAGACTTGCAGAGCGGCTTTTTTG |
|  | R | AATTCAAAAAAGCCGCTCTGCAAGTCTAATACTCGAGTATTAGACTTGCAGAGCGGCT |
| mouse Tfap2c (shT2) | F | CCGGCCACGTCGAAGTACAAAGTAACTCGAGTTACTTTGTACTTCGACGTGGTTTTTG |
|  | R | AATTCAAAAACCACGTCGAAGTACAAAGTAACTCGAG TTACTTTGTACTTCGACGTGG |
| mouse Cdh1  (shC1) | F | CCGGGGTGAAGGCTTGAGCACAACACTCGAGTGTTGTGCTCAAGCCTTCACCTTTTTG |
|  | R | AATTCAAAAAGGTGAAGGCTTGAGCACAACACTCGAGTGTTGTGCTCAAGCCTTCACC |
| mouse Cdh1  (shC2) | F | CCGGGCAGCAATACATCCTTCATGTCTCGAGACATGAAGGATGTATTGCTGCTTTTTG |
|  | R | AATTCAAAAAGCAGCAATACATCCTTCATGTCTCGAGACATGAAGGATGTATTGCTGC |
