# Supplementary material for: TFAP2C facilitates somatic cell reprogramming by inhibiting c-Myc-dependent apoptosis and promoting mesenchymal-to-epithelial transition
Source: Cell Death Dis. 2020 Jun 25;11(6):482. doi: 10.1038/s41419-020-2684-9 (PMC7316975; doi:10.1038/s41419-020-2684-9)
Supplement: Supplementary file 14 — Supplementary Table 2 [file 41419_2020_2684_MOESM14_ESM.docx]

| **Genes that may inhibit reprogramming and downregulated simultaneously in our RNA-seq dataset** | | **Genes that may facilitate reprogramming and upregulated simultaneously in our RNA-seq dataset** | |
| --- | --- | --- | --- |
| **day 4** | **day 8** | **day 4** | **day 8** |
| Jag2 | Vax2 | Prl3d3 | Prl3d3 |
| Prrx1 | Prrx1 | Hand1 | Htr1d |
| Gli2 | Col6a2 | Esrrb | Dazl |
| Col6a2 | Jag2 | Lin28a | Hand1 |
| Bmp2 | Scg5 | Aldh1a3 | Dppa4 |
| Cacna1g | Cacna1g | Dppa4 | Esrrb |
| Meox2 | Bmp2 | Lpar5 | Dppa2 |
| Tead2 | Meox2 | Wnt7b | Serpinb6b |
|  | Tead2 | Tdgf1 | Zfp42 |
|  |  | Utf1 | Mx2 |
|  |  | Cldn4 | Apol9b |
|  |  | Prlr | Lair1 |
|  |  | Il2rg | Prlr |
|  |  | Wnt9a | Lin28a |
|  |  | St8sia1 | Utf1 |
|  |  | Zfp42 | Tdgf1 |
|  |  | Sema7a | Sfmbt2 |
|  |  | Serpinb6b | Mdk |
|  |  | Crb3 | Wnt9a |
|  |  | Eps8l2 | Smtn |
|  |  | Sema3e | Ddx58 |
|  |  | Tnfaip2 | F3 |
|  |  | Cldn7 | Wnt7b |
|  |  | Irf6 | Nodal |
|  |  | Cdh1 | Il2rg |
|  |  | Lck | Tcf7 |
|  |  | Barx2 | Ass1 |
|  |  | Sall4 | Oasl1 |
|  |  | F3 | Lck |
|  |  | Ass1 | Sema3f |
|  |  | Ddx58 | Sema3e |
|  |  | Nodal | Sall4 |
|  |  | Epcam | Cldn4 |
|  |  | Parvb | Aldh1a3 |
|  |  | Esrp1 | Cib2 |
|  |  | Cldn3 | St8sia1 |
|  |  | Dsg2 | Gdf3 |
|  |  | Fam71f1 | Kitl |
|  |  | Ehhadh | Sync |
|  |  | Cd34 | Tpm2 |
|  |  | Fgd6 | Cdh1 |
|  |  | Sfmbt2 |  |
|  |  | Tpm2 |  |
|  |  | Ier3 |  |
|  |  | Ocln |  |
|  |  | Scnn1a |  |
|  |  | Hmgxb4 |  |
|  |  | Tpd52 |  |
|  |  | Slc16a13 |  |
|  |  | Wipi1 |  |
