# Supplementary material for: TFAP2C facilitates somatic cell reprogramming by inhibiting c-Myc-dependent apoptosis and promoting mesenchymal-to-epithelial transition
Source: Cell Death Dis. 2020 Jun 25;11(6):482. doi: 10.1038/s41419-020-2684-9 (PMC7316975; doi:10.1038/s41419-020-2684-9)
Supplement: Supplementary file 15 — Supplementary Table 3 [file 41419_2020_2684_MOESM15_ESM.docx]

| **Term** | **Gene ↓** | **Gene ↑** |
| --- | --- | --- |
| Apoptosis （Day4） | Trp53; p14ARF; Fas; Bax; PTEN; Bid; CASP3; Cycs; Cdkn2a; Casp2; Bid; Bax; Fadd; Map3k5; Casp7; Kcnma1; Tgm2; Bmp2; Sfrp1; Cnr1; Rassf2 | Foxo3; bcl-xl; Gadd45a; Bcl2l1; Foxo1; Tgfa; Bcl3; Bcl2l2; Ptgs2; Igf2r; Gadd45b; Bcl10 |
| Apoptosis （Day8） | p14ARF; p21; Bax; PUMA; Bid; CASP3; Bdnf; Alox12; Rps6ka2; Epha7; Cycs; Cyclin D; Rassf2 | Bcl2l1; Bcl3; bcl-xl; Gadd45a; ptpn13; birc3; Mmp9; Cited2; Ctnna1 |
